# The Duality of the Mind Proved by the Structure, Functions, and Diseases of the Brain, &c.

**Published:** 1845-04

**Authors:** 


### The Duality of the Mind proved by the Structure, Functions, and Diseases of the Brain, and by the Phenomena of Mental Derangement, and shewn to be Essential to Moral Responsibility. With an Appendix: 1. On the Influence of Religion on Insanity. 2. Conjectures on the Nature of the Mental Operations 3. On the Management of Lunatic Asylums. By *A. L. Wigan*, M.D. 8vo. pp. 459. London: Longmans, 1844.

Dr. Wigan's work, whatever may be thought of its other merits, may at least claim that of novelty; indeed, we do not recollect any production of late years, during which a more cautious spirit has been introduced into philosophical inquiries, displaying so much speculation, and that of the most startling kind. This "New View of Insanity" also evinces much acute observation, combined with a benevolent spirit, and many of the instances and illustrations adduced by the author in support of his theory are plausible and ingenious. But, in readily admitting the just claims of Dr. Wigan, we confess that we have finished the perusal of this work sceptical as to his conclusions, or rather, to speak more plainly, with the conviction that the hypothesis of the Duality of the Mind, as presented to public notice in the treatise before us, is an entire fallacy. The author observes in the Introduction:

"The unsettled state of mental philosophy in the present day is such as to justify any man in the endeavour to throw light on a subject so dark and intricate, more especially if the discovery of a new instrument seem to give new and extraordinary facilities for the investigation. Physiological reasoning could advance but a little way till the circulation of the blood was ascertained; and whatever may be the degree of merit awarded to myself on the present occasion, I cannot but think that I bring to the investigation of the mental phenomena in health and disease a new power, of at least equal importance, and capable of equally satisfactory demonstration." P. 2.

Coinciding with the remarks contained in the former part of this paragraph, we regret that it is not in our power to admit either the degree of importance claimed for the doctrine of the dual mind, or the satisfactoriness of the demonstration by which the attempt is made to support it. And we make this last remark with no desire to disparage the labours of Dr. Wigan, for the truth is, that the subject is not susceptible of the kind of proof by which Harvey did in reality *demonstrate* the course of the blood. The regular and definite propulsive actions distinctly seen by observing the heart of the living reptile; the mode of attachment and obvious mechanism of the valves contained in the veins and at the origin of the aorta and pulmonary artery; the turgidity caused on the distal side of the ligature when a vein is tied and on the cardiac side in the case of an artery; the hemorrhage which in the instance of a wounded artery proceeds from the end towards the heart, but which from an injured vein proceeds from the remote extremity; these, it is apparent, are facts of a totally different kind from any which can be obtained in the investigation of the mind. This passing notice of the facts which established the true theory of the circulation, forces upon us the recollection that there is indeed one discovery of modern times which may strictly be compared with that of the illustrious Harvey—it is that of Bell, who, by his profoundly philosophic researches, and by demonstrating the course of the nervous currents, accomplished for the nervous system what his great predecessor effected for the vascular.

We have further to remark that, in those parts of the argument where anatomical and physiological proofs or illustrations are adduced, the author is particularly unfortunate in his selections. At page 36, for example, the following passage occurs: "The ganglionic, or great sympathetic system of *little brains* in the interior of the body, connected by a network of nerves (like the additional spring to a watch, to enable it to go while winding up), carries on the functions of life during sleep, while the action of the cerebral organs is suspended." And again (p. 143) in alluding to the fact that, the pressure of the finger upon the brain of an animal in a physiological experiment causes coma, it is stated, "the body remains a mere lump of organized matter dependent on the ganglionic system for its vitality." These propositions embody a profound physiological error; so profound indeed, that it naturally excites a doubt, if any one entertaining such crude notions, can be qualified to undertake an inquiry of the most subtle character and involving the highest functions of the nervous system. Setting aside all the uncertainty which at this time prevails respecting the powers of the great sympathetic in maintaining the circulation, no one who from the time of Le Gallois to Marshall Hall has investigated the subject, has any doubt that during sleep and in the comatose condition, as at all other times, the vital motions of respiration are essentially dependent on, and are sustained by, the medulla oblongata and the upper part of the spinal cord, operating through the pneumo-gastric and phrenic nerves.

Without, however, dwelling in this place longer upon the impressions excited in our own mind by Dr. Wigan's treatise, we will lay before our readers sufficient extracts to enable them to judge for themselves, a mode of procedure which, where there is so much novelty and so much speculation, will be most fair towards the author and most satisfactory to ourselves. Dr. Wigan's hypothesis is thus explained :—

"Entertaining no doubt whatever that every candid man, after reading these remarks, will at least adopt my nomenclature, I shall in future speak of the two cerebra instead of the two hemispheres; being certain that I shall prove the propriety and utility, nay, the absolute necessity, of using the former term instead of the latter, which has led (as I shall shew) to false inferences, and has no advantage whatever to counterbalance the mischief.

I believe myself then able to prove—

1. That each cerebrum is a distinct and perfect whole, as an organ of thought.
2. That a separate and distinct process of thinking or ratiocination may be carried on in each cerebrum simultaneously.
3. That each cerebrum is capable of a distinct and separate volition, and that these are very often opposing volitions.
4. That, in the healthy brain, one of the cerebra is almost always superior in power to the other, and capable of exercising control over the volitions of its fellow, and of preventing them from passing into acts, or from being manifested to others.
5. That when one of these cerebra becomes the subject of functional disorder, or of positive change of structure, of such a kind as to vitiate mind or induce insanity, the healthy organ can still, up to a certain point, control the morbid volitions of its fellow.

6. That this point depends partly on the extent of the disease or disorder, and partly on the degree of cultivation of the general brain in the art of self-government.
7. That when the disease or disorder of one cerebrum becomes sufficiently aggravated to defy the control of the other, the case is then one of the commonest forms of mental derangement or insanity; and that a lesser degree of discrepancy between the functions of the two cerebra constitutes the state of conscious delusion.
8. That in the insane, it is almost always possible to trace the intermixture of two synchronous trains of thought, and that it is the irregularly alternate utterance of portions of these two trains of thought which constitutes incoherence.
9. That of the two distinct simultaneous trains of thought, one may be rational and the other irrational, or both may be irrational; but that, in either case, the effect is the same, to deprive the discourse of coherence or congruity.

Even in furious mania, this double process may be generally perceived; often it takes the form of a colloquy between the diseased mind and the healthy one, and sometimes even resembles the steady continuous argument or narrative of a sane man, more or less frequently interrupted by a madman; but persevering with tenacity of purpose in the endeavour to overpower the intruder.

10. That when both cerebra are the subjects of disease, which is not of remittent periodicity, there are no lucid intervals, no attempt at self-control, and no means of promoting the cure; and that a spontaneous cure is rarely to be expected in such cases.
11. That however, where such mental derangement depends on inflammation, fever, gout, impoverished or diseased blood, or manifest bodily disease, it may often be cured by curing the malady which gave rise to it.
12. That in cases of insanity, not depending on structural injury, in which the patients retain the partial use of reason (from one of the cerebra remaining healthy or only slightly affected), the only mode in which the medical art can promote the cure beyond the means alluded to, is by presenting motives of encouragement to the sound brain to exercise and strengthen its control over the unsound brain.
13. That the power of the higher organs of the intellect to coerce the mere instincts and propensities, as well as the power of one cerebrum to control the volitions of the other, may be indefinitely increased by exercise and moral cultivation; may be partially or wholly lost by desuetude or neglect; or, from depraved habits and criminal indulgence in childhood, and a general vicious education in a polluted moral atmosphere, may never have been acquired.
14. That one cerebrum may be entirely destroyed by disease, cancer, softening, atrophy, or absorption; may be *annihilated*, and in its place a yawning chasm; yet the mind remain complete and capable of exercising its functions in the same manner and to the same extent that one eye is capable of exercising the faculty of vision when its fellow is injured or destroyed; although there are some exercises of the brain, as of the eye, which are better performed with two organs than one. In the case of vision, the power of measuring distances for example, and in the case of the brain, the power of concentrating the thoughts upon one subject, deep consideration, hard study; but in this latter case, it is difficult to decide how far the diminished power depends on diminution of general vigour from formidable and necessarily fatal disease.
15. That a lesion or injury of both cerebra is incompatible with such an exercise of the intellectual functions, as the common sense of mankind would designate *sound mind.*

16. That from the apparent division of each cerebrum into three lobes, it is a natural and reasonable presumption that the three portions have distinct offices, and highly probable that the three great divisions of the mental functions laid down by phrenologists, are founded in nature; whether these distinctions correspond with the natural divisions is a different question, but the fact of different portions of the brain executing different functions, is too well established to admit of denial from any physiologist.

17. That it is an error to suppose the two sides of the cranium to be always alike, that on the contrary, it is rarely found that the two halves of the exterior surface exactly correspond; that indeed, in the insane, there is often a notable difference—still more frequent in idiots, and especially in congenital idiots.

18. That the object and effect of a well-managed education are to establish and confirm the power of concentrating the energies of both brains on the same subject at the same time; that is, to make both cerebra carry on the same train of thought together, as the object of moral discipline is to strengthen the power of self-control; not merely the power of both intellectual organs to govern the animal propensities and passions, but the intellectual antagonism of the two brains, each (so to speak) a sentinel and security for the other while both are healthy; and the healthy one to correct and control the erroneous judgments of it fellow when disordered.

19. That it is the exercise of this power of compelling the combined attention of both brains to the same object, till it becomes easy and habitual, that constitutes the great superiority of the disciplined scholar over the self-educated man; the latter may perhaps possess a greater stock of useful knowledge, but set him to study a new subject, and he is soon outstripped by the other, who has acquired the very difficult accomplishment of *thinking of only one thing at a time;* that is, of concentrating the action of both brains on the same subject.

20. That every man is, in his own person, conscious of two volitions, and very often conflicting volitions, quite distinct from the government of the passions by the intellect; a consciousness so universal, that it enters into all figurative language on the moral feelings and sentiments, has been enlisted into the service of every religion, and forms the basis of some of them, as the Manichæan." P. 30.

The author frequently adduces the provision of two eyes and two ears, as an argument not only for the provision of " two distinct and perfect brains," but also of two minds. In connexion with this subject the author, in the following passage, has been led by his favourite theory into what, as it seems to us, is a palpable error: A gentleman, owing to an injury of the head, for a short time lost his memory and power of articulation, and although these were soon restored, two days subsequently " he heard two voices close to his ear, in rapid dialogue, almost without meaning. When reading, similar voices seemed to accompany him, sometimes getting a few words in advance, but not beyond what the eye might have reached. *I call this one brain reading faster than the other.*" Without dwelling upon the looseness of this conclusion in other respects, we would remark that it involves a physiological impossibility, for, as the impressions from the book must have been conveyed by the two optic nerves to the brain at precisely the same instant, the perceptions, even allowing them to have been double, must have corresponded *as to time.*

But the question of duplex *organs*, is totally different from that which relates to a double power of *perception*—of *comparison*—of *judgment*; so that the brain may be, as indeed it frequently has already been considered, as double *quoad* organs, but not as concerns functions. And we may further remark, that in order to associate the double organs composing the cerebrum, and to convert them, as to action, into *one instrument*, the transverse commissures, as they are termed, are provided, and so elaborately are they developed that it is probable every individual part of the brain on one side, is brought into connexion with the corresponding part of the other side. Now, in the case of the double organs, such as the eye, the ear, the two arms, and so forth, which do act singly, no such organic connexion is established; it is met with only in the organ of the mind. In alluding to a case which had been pointed out as opposed to his theory, Dr. Wigan, in speaking of the corpus callosum, says, the case "fully confirms my opinion that it is an organ of no importance, and not necessary to the functions of the brain." Those who have traced the extent and relations of this, the largest body in the brain, or who have studied the accurate plates of Mayo, Solly, and Arnold, will scarcely admit the probability of this opinion, nor will they place much faith in a doctrine which requires for its support such an extraordinary assumption. It is another of Dr. Wigan's positions, that "one of the cerebra is almost always superior to the other;" no proof of this is, however, given, as far as we can perceive, beyond the bare fact, to which no physiologist would attach any importance as an argument of this kind, that inasmuch as the right arm and right leg are more powerful than the left, it is to be inferred that the left brain acts more energetically than its opposite fellow.

A great number of cases, some consisting of those slighter forms of delusions, which, although they never occur in the perfectly healthy mind, do not amount to insanity, and others of decided insanity, are adduced in support of the theory before us. One or two of these cases will, in connexion with the above extracts, illustrate the author's train of argument.

We have the following explanation of those day-dreams in which all of us have at some time or other indulged, and which we therefore submit as a kind of typical embodying of Dr. Wigan's views.

"The well-known process called castle-building—in which some persons can indulge till the false impression shall influence their actions—seems to me explicable in the same manner. One brain is allowed to go on with a train of thought which produces pleasurable sensations, unchecked by the conscious other, till the effort required to stop the disordered process becomes difficult or impossible. While the volition remains omnipotent, the case is merely the power of dwelling exclusively on pleasurable ideas—of which hope is one of the forms—the further indulgence of the habit produces almost the effect of truth, and the dull realities of life become insipid." P. 118.

A case of mental delusion is related of a man who was convinced that he was haunted by a kind of *second self*. This alter ego "would argue with him pertinaceously, and to his great mortification sometimes refute him, which, as he was very proud of his logical powers, humiliated him exceedingly." "In sitting by his side, I sometimes heard him exclaim 'well, that takes me quite aback, I must consider a little for an answer.'" "I know not what effect such an example might produce on others, but to me it seems only to be explained on the hypothesis of two brains with distinct and contradictory trains of thought at the same time. The inference seems irresistible that, with conflicting volitions and conflicting trains of thought, there are in the disordered cerebrum two perfect and complete organs of the understanding in habitual antagonism, and that uniformity of *will*, when consentaneity does not exist, is produced by the tyranny of one brain over the other." (P. 127).

"In summing up what has been said, I think it may be assumed without risk of contradiction, that the fact of each brain being a perfect and complete instrument of thought is abundantly proved. That each, while in health, corresponds entirely in action with its fellow, is obvious from the fact that this unison and correspondence give only one result, as in the case of the two eyes producing single vision. That when from any cause one brain is disordered, a discrepancy in the two processes of thinking takes place. That the healthy brain (aided by the action of such of the organs of its fellow as are not affected by the disorder which disturbs the others) can, in nearly nine hundred and ninety-nine cases in a thousand, according to the usual proportion in this country, control all manifestation of morbid emotion or judgment, but that the thousandth case is the madman." P. 275.

These extracts will enable our readers to comprehend the theory advanced by Dr. Wigan. For ourselves, we must re-iterate our dissent, as it seems to us that all these and similar cases can be satisfactorily explained by regarding them as so many different forms and degrees of disturbance affecting the faculties of a single consciousness. No proof is given in these mental hallucinations of the real and independent existence of two intellectual entities ; on the contrary, as the author himself states, there are in most cases of insanity, where this double train of thought is going on, occasional lucid intervals, in which the unhappy patient is conscious of the deceit and of his own individuality.

Although we cannot accede to the peculiar views of Dr. Wigan, we most willingly bear our testimony to the philanthropic spirit pervading the whole treatise.

His observations on the causes of insanity are also most just, and are worthy of the serious consideration of all who, whether professionally or otherwise, are called upon to minister to the insane, the most afflicted class of our fellow-beings. The fact that every form of mental derangement depends upon some form or other of cerebral disease—and by this term we signify every, the least, departure from the standard of health, is at length pretty generally admitted ; but notwithstanding this, the remarks we are about to make are so apposite that they will not be misplaced.

"The word *insanity* means *un-health*, and it means nothing more. Why the term should have been applied to the class of diseases which, by disordering the brain, disturb the intellect, I know not; but the consequences of such restriction of its meaning have been mischievous. We are set to seek the causes of insanity as if it were *a disease*, whereas it is the effect or result of many diseases. The physician is expected to cure a disorder of the *mind*, as if it were something quite distinct from a disorder of the body. This is exactly equivalent to the step of which I have elsewhere spoken, of addressing a watchmaker thus: 'Here is my watch—the motion is wrong, and I have brought it to you to be put right; but you must not touch the works—all the wheels, pivots, springs, balances, verge, contract pinion, and so forth, are quite in order. I do not know the structure of a watch, for I have never taken one to pieces or put it together, but have read books on the subject, and from the knowledge thus acquired, am sure that there is nothing wrong in the machinery of that I am now putting into your hands; therefore I beg you will not touch the works, but merely set right *the motion*, for that is the only defect.'" P. 117.

In considering *the forms* of mental derangement, we feel it due to the science of Phrenology to express our conviction that the four great classes of intellectual phenomena—the propensities, the sentiments, the perceptive, and the reflective faculties, contended for by Spurzheim and his school, have a real existence; but in making this admission, we are by no means satisfied of the 35 subdivisions, and as to that which constitutes the peculiar part of this doctrine, the localization, namely, of those subdivisions in certain definite convolutions of the brain, we hold it not proved.

The adoption of so much of the phrenological system, and this is the amount of belief accorded to it by Dr. Wigan, will enable us to form clear conceptions respecting the various types of mental disturbance, and how it happens that one class of phenomena may remain intact, whilst another class is utterly deranged. The best writers upon this subject have marked these distinctions. Thus, to say nothing of monomania, in which there is illusion upon one particular subject only, we have that most prevailing species of disorder which has been well named "moral insanity," in which, as Esquirol remarks, although the passions and moral affections are perverted or destroyed, it may be difficult or impossible to trace any hallucination or disturbance of the perceptive and reflective faculties. The great frequency of this form of derangement, which is in fact much more prevalent than any other type, indicates the importance of considering how the tendency towards it may be controlled or prevented; there is, indeed, no question which more nearly concerns the well-being and happiness of society, nor one in which our profession, by inculcating right views, can more effectively benefit the community. A pamphlet having for its title, "On Man's Power over himself to prevent or control Insanity," by the Rev. J. Barlow, Secretary of the Royal Institution, contains some excellent observations upon this subject; the principal position contended for being "that the difference between sanity and insanity consists in the degree of self-control exercised." Excluding those cases which depend upon *physical* causes, though even a large number of this class is produced by vicious habits,* it is apparent that a considerable number of the cases met with in every asylum may be traced to a want of that rigid discipline of the mind, which is one of the most difficult, and yet one of the most important of the moral lessons, which man has to acquire on this side of the grave. Domestic griefs, reverses of fortune, jealousy, injured self-love, religious enthusiasm, what are these, the potential "moral causes" of insanity, but so many trials which come to all of us, and which, if not moderated by firmness, by humility, and above all, by the pervading conviction of the uncertain tenure of all earthly happiness, will overturn the throne of reason. Mr. Barlow well observes, "should my position, that the difference between sanity and insanity consists in the degree of self-control exercised, appear paradoxical to any one, let him note for a

---

* For example, out of 256 cases dependent upon physical causes, as investigated by M. Esquirol at the Maison Royale de Charenton, 127, or more than half, were produced by causes resulting from defective moral control; Masturbation 23, Libertinism 24, Use of Mercury 16, Abuse of Wine 64.

short time the thoughts that pass through his mind, and the feelings that agitate him; and he will find that, were they all expressed and indulged, they would be as wild, and perhaps as frightful in their consequences, as those of any madman. But the man of strong mind represses them, and seeks fresh impressions from without, if he finds that aid needful: the man of weak mind yields to them, and then he is insane."

The experience of the best observers confirms this view of the subject. How important are these remarks of Dr. Conolly: "seeing that any feeling in excess—the love of pleasure, or of ease, or of money, or of expense, or of applause; or that self-denial, or anger, or jealousy, or hope too sanguine, or sorrow too much indulged—may become independent of the restraint of the comparing powers, and thus impair or disorder the understanding, we cannot but remark the importance of cherishing that governing and protecting action of the mind by careful cultivation and exercise." "Whoever will converse with lunatics, will soon be satisfied that a very small portion of them consists of persons whose talents have been regularly and judiciously cultivated."

A knowledge of the evils arising from this want of self-control is indispensable to the right treatment of insanity; for, although it is indeed a difficult task to awaken those better feelings which have long slumbered, or, which is worse still, have never been acquired, the almost unhoped-for success which has attended the non-restraint system, and its concomitant adjuncts, mental and bodily occupation, is all-sufficient proof that intelligence and zeal, combined with benevolence and patience, will, in the end, triumphing over all obstacles, be rewarded with the happiest results.

We must here conclude our notice of Dr. Wigan's "New View of Insanity," which, without departing from the opinion we have expressed of its fallaciousness, we commend to the notice of our readers, as worthy of perusal, on account both of the great interest of the subject, and of the ingenuity evinced by the author.

